# Academic mentoring in nursing as communicative praxis and existential formation

**DOI:** 10.15649/cuidarte.5658

**Published:** 2026-08-14

**Authors:** Montserrat Mariscal Delgadillo

**Affiliations:** 1 University of Guadalajara, University Center for Health Sciences (CUCS), Department of Applied Clinical Nursing, Jalisco, Mexico. E-mail: montserrat.mariscalde@academicos.udg.mx University of Guadalajara Jalisco Mexico montserrat.mariscalde@academicos.udg.mx

**Keywords:** Critical Theory, Existentialism, Mentoring, Health Sciences, Nursing, Teoría Crítica, Existencialismo, Tutoría, Ciencias de la Salud, Enfermería, Teoria Crítica, Existencialismo, Tutoria, Ciências da Saúde, Enfermagem

## Abstract

**Introduction:**

Academic mentoring is a central strategy in nursing education, as it influences student retention and the quality of the educational process; however, implementing mentoring presents conceptual and operational ambiguities that limit its scope.

**Objective:**

To analyze mentoring through Habermas’s Theory of Communicative Action and Kierkegaard’s existential perspective, in order to make visible its dialogical, ethical, and subjective dimensions.

**Content Synthesis:**

This reflection article is based on a review and integration of philosophical, pedagogical, and disciplinary literature on academic mentoring in nursing, in dialogue with findings from previous empirical research on teaching competencies and challenges in mentoring practice in the Mexican context, which serve as problematizing points of reference.

**Conclusion:**

Mentoring extends beyond the logics of academic incentives and compliance with educational quality indicators and is, therefore, understood as an ethical and meaningful practice that requires not only normative and pedagogical clarity but also the integration of communicative rationality, existential guidance, and professional responsibility.

## Resumen

**Introducción:**

La tutoría académica constituye una estrategia central en la formación de estudiantes de Enfermería, al incidir en la permanencia estudiantil y en la calidad del proceso formativo; sin embargo, su implementación presenta ambigüedades conceptuales y operativas que limitan su alcance.

**Objetivo:**

Analizar la tutoría desde la Teoría de la Acción Comunicativa de Habermas y la perspectiva existencial de Kierkegaard, a fin de visibilizar sus dimensiones dialógicas, éticas y subjetivas.

**Síntesis de Contenido:**

Artículo de reflexión crítica construido a partir de la revisión e integración de la literatura filosófica, pedagógica y disciplinar sobre la tutoría académica en Enfermería, articulada con hallazgos de investigaciones empíricas previas sobre competencias docentes y desafíos en la acción tutorial en el contexto mexicano, utilizados como referentes problematizadores.

**Conclusiones:**

La tutoría trasciende las lógicas del estímulo académico y del cumplimiento de indicadores de calidad educativa, siendo entendida, por lo tanto, como una práctica ética y con sentido que no solo requiere claridad normativa y pedagógica, sino también la integración de racionalidad comunicativa, acompañamiento existencial y responsabilidad profesional.

## Resumo

**Introdução:**

A tutoria acadêmica é uma estratégia central na formação de estudantes de enfermagem, influenciando a retenção dos alunos e a qualidade do processo educativo; contudo, sua implementação apresenta ambiguidades conceituais e operacionais que limitam seu alcance.

**Objetivo:**

Analisar a tutoria a partir da Teoria da Ação Comunicativa de Habermas e da perspectiva existencial de Kierkegaard, a fim de destacar suas dimensões dialógica, ética e subjetiva.

**Síntese do conteúdo:**

Este artigo de reflexão crítica baseia-se em uma revisão e integração da literatura filosófica, pedagógica e disciplinar sobre tutoria acadêmica em enfermagem, articulada com achados de pesquisas empíricas anteriores sobre competências docentes e desafios da tutoria no contexto mexicano, utilizados como referências problematizadoras.

**Conclusões:**

A tutoria transcende a lógica da estimulação acadêmica e do cumprimento de indicadores de qualidade educacional, sendo entendida, portanto, como uma prática ética e significativa que requer não apenas clareza normativa e pedagógica, mas também a integração da racionalidade comunicativa, do acompanhamento existencial e da responsabilidade profissional.

## Introduction

Health sciences education (HSE) in the twenty-first century faces the need to respond to the growing technoscientific complexities of human care without relinquishing the human and ethical dimensions of health professions. In this regard, academic mentoring has been established, in both international discourse and national and local frameworks, as a leading educational strategy^1^-^4^. Mentoring is recognized for its decisive role in improving educational quality, preventing dropout and educational lag^5^,^6^ and promoting comprehensive education for students^7^.

In this context, the 2030 Agenda for Sustainable Development, in Goal 4, calls for ensuring inclusive, equitable, and quality education, as well as promoting lifelong learning opportunities for all^8^. Target 4.3 seeks to ensure equal access to tertiary education, while Target 4.7 promotes the acquisition of knowledge and competencies that advance sustainable development, gender equality, human rights, and a culture of peace^8^. Accordingly, academic mentoring emerges as a tool that extends beyond academic support and is linked to equity, personal development, and the social commitment of future health professionals.

This horizon almost naturally connects with the four pillars identified by Delors: learning to know, learning to do, learning to live together, and learning to be. In mentoring practice, these dimensions take on specific forms: learning to know is expressed in fostering critical and reflective thinking; learning to do, in guiding ethical professional practice; learning to live together, in sustaining dialogic, respectful, and collaborative relationships; and learning to be, in promoting the individual’s holistic development, which in nursing is reflected in the development of a humanistic, self-aware professional identity committed to human care^9^.

This estimation coexists with conceptual polysemy and heterogeneity in practice^10^,^11^; and in the absence of a clear delineation of its meaning, mentoring risks being reduced to administrative follow-up, academic monitoring, or even to just another competency, thereby overlooking its transformative potential^12^,^13^.

In nursing higher education, academic mentoring has been progressively institutionalized as a support program aimed at assisting students’ educational trajectories, particularly at critical points in the educational process. These programs are typically structured around regulatory guidelines that assign mentors responsibilities in academic guidance, academic monitoring, and institutional referral; however, they often lack an explicit pedagogical and philosophical foundation that integrates these functions within a comprehensive educational framework. This condition has led to heterogeneous mentoring practices, shaped by diverse interpretations of its purpose, scope, and meaning in professional nursing education^4^,^5^.

This contrasts with findings from a previous study^12^, which examined the relationship between teaching competencies and mentoring-related challenges in the Mexican context. These findings revealed ambiguities arising, on the one hand, from the lack of a clear delineation of the mentor’s role across academic, administrative, and formative functions, and, on the other, from the coexistence of approaches that conceptualize mentoring either as institutional monitoring of students’ academic trajectories or as comprehensive student support. These ambiguities prompt the following questions: What is the true meaning of mentoring in nursing education? Is its scope limited to enhancing academic trajectories, or does it point toward a deeper horizon, linked to holistic human development, ethical awareness, and the existential formation of professional identity?

Addressing these questions requires moving beyond technical-administrative frameworks, as the empirical-analytical logic, while necessary, often obscures the intersubjective dimensions of the educational process^14^. Hence, a philosophical approach is needed to conceptualize mentoring as a complex human phenomenon. Accordingly, this reflection engages in dialogue with two key thinkers: Jürgen Habermas, whose notion of communicative action and dialogical rationality provides a framework for understanding mentoring as a space of mutual understanding and emancipation^15^,^16^ and Søren Kierkegaard, whose reflections on subjectivity, authenticity, and freedom reposition mentoring within the existential dimension of responsibility^17^,^18^.

Per se, by integrating both perspectives, mentoring is conceived not only as academic support, but as humanistic praxis; a formative space in which professional competence and existential awareness converge, guiding the preparation of professionals who are not only skilled, but also ethically grounded and responsible for the care of human life. This reflection article, therefore, draws on a critical review of philosophical, pedagogical, and nursing literature, conducted using specialized academic sources selected for their theoretical relevance and pertinence to the field of nursing education, complemented by findings from prior empirical research on teaching competencies and academic mentoring in undergraduate nursing^12^, integrated through an interpretive and problematizing dialogue.

## Content Synthesis

**Mentoring as a space of communicative action in the education of nursing human resources**

Approaching academic mentoring from Jürgen Habermas’s Theory of Communicative Action entails shifting its understanding from a technical-instrumental model toward an intersubjective horizon, one in which dialogue becomes the structuring core of the pedagogical relationship, and in which mentor and student engage in an interaction free from coercion, grounded in mutual recognition and a shared search for meaning^19^-^21^.

In this sense, the mentoring encounter acquires ethical and epistemic depth; rather than a simple transmission of content, it is constituted as a discursive practice in which the values, rules, and contexts that shape nursing are critically examined^22^. Such praxis is not limited to student performance; it is projected as a catalyst for critical consciousness capable of challenging the normative frameworks that configure knowledge and power in the field of the health sciences^14^,^16^. Mentoring calls forth not only the educator’s competence but also an ethical disposition to foster conditions for symmetrical dialogue; Habermasian thought makes it possible to situate this practice within the historicity of educational processes, recognizing that nursing education unfolds within social and institutional structures that reproduce exclusion, hierarchy and inequality^23^,^24^. Mentoring, however, can become a space of formative resistance and for the shaping of emancipatory subjectivities when it is grounded in the unconditional recognition of the other’s dignity^21^,^22^.

From this perspective, Delors’s pillars of education^9^ find an experiential correlate: in mentoring practice, learning to know is enacted through reflective dialogue in Habermasian terms, entailing an orientation of action toward mutual understanding^15^. Truth, in this sense, is validated through processes of argumentation free from coercion. In turn, learning to do is realized through critical mediation between knowledge and situated action^19^.

Learning to live together is experienced when mentoring promotes mutual legitimacy and fosters equitable pedagogical relationships; this points to the social dimension of language, in which participants recognize one another as interlocutors within a community. Finally, learning to be occurs as the student is constituted as a moral and discursive agent through the recognition of their voice, their life project, and their right to make meaning of the world^15^. Communicative action thus fosters mentoring oriented toward critical and ethical formation, capable of transforming nursing students into subjects who deliberate, act, and live together in complex societies, thereby positioning mentoring not only as a pedagogical act but also as an ontological one^14^,^20^.

**Academic mentoring as existential guidance and professional responsibility**

Beyond its communicative dimension, mentoring can be understood as existential guidance; that is, as a process oriented toward the ethical constitution of the subject being prepared to provide human care. From this perspective, Kierkegaard’s perspective is particularly relevant, as it shifts the emphasis from the mere acquisition of objective knowledge to the development of authentic subjectivity, inner freedom, and personal responsibility^25^,^26^. Mentoring, therefore, ceases to be a transmissive function and becomes a formative mediation; the mentor is no longer presented as a repository of certainties, but as one who calls existence into question.

In line with Kierkegaard’s concept of “subjective truth,” mentoring is oriented toward enabling the student to integrate knowledge into the totality of their lived experience^18^. This integration is not achieved through doctrinal imposition but through what Kierkegaard termed “indirect communication,” a pedagogical mode in which the mentor, per se, acts as a mediator who elicits critical reflection and confronts illusions, fears, and hopes^24^,^25^. Mentoring does not neutralize the anxiety of professional becoming; rather, it brings it to the fore, inviting its recognition as a constitutive dimension of care and encouraging the becoming of the self capable of responding ethically to the uncertainty inherent in every professional action^27^,^28^.

Mentoring becomes a space in which the student lives in accordance with what they recognize as true. This “reduplication,” according to Kierkegaard, entails the fusion of existence and conviction^27^. It is not merely a matter of acquiring clinical skills; rather, it involves assuming responsibility for being in the world of care with authenticity, that is, with a consciousness that does not delegate its freedom to protocols or its judgment to external norms. Mentoring, however, presents knowledge not as fixed instruction but as a skill, whose appropriation requires sustained attentiveness and inner responsiveness to the irreducible complexity of the other^26^,^18^.

Analogous to the foregoing discussion of Habermas’s communicative rationality, Kierkegaardian existentialism may be understood as another philosophical key for re-signifying academic mentoring. Whereas, within the Habermasian framework, mentoring is conceived as an intersubjective praxis oriented toward mutual understanding and emancipation^15^,^19^, in Kierkegaard’s perspective, mentoring is interpreted as a radical form of guidance that calls the student to confront the meaning of their professional existence^25^,^28^. It is not, therefore, a matter of prescribing a way of being; rather, it entails enabling the subject to recognize themselves as an ethical agent amid the uncertainty of care, articulating freedom and responsibility in the way they inhabit their vocation^28^,^26^.

In doing so, the formative aims underlying the pillars of education9 are reinterpreted through lived subjectivity, not as normative mandates, but as existential possibilities within the educational trajectory of future nursing professionals.

**The meaning of mentoring, the formative ideal, and the institutional context**

As discussed in this article, mentoring in university nursing education cannot be reduced to a technical dimension or an administrative process; at its core, it is a formative vocation whose meaning extends beyond academic guidance and is situated within an ethical, reflective, and ontological framework. In its deepest expression, mentoring and teaching are intertwined as ways of inhabiting the world of health sciences education, oriented toward the formation of ethically committed professional subjectivities^14^.

From the Theory of Communicative Action, the educator is an agent of dialogical rationality: their task is to foster mutual understanding, cultivate communicative competencies, and open deliberative spaces in which students critically question both knowledge and its social embeddedness^15^,^19^,^16^. This perspective deepens when brought into dialogue with Kierkegaard’s existential philosophy; here, the educational act finds its meaning in guiding toward authenticity. The educator, therefore, does not instruct through control but guides with discretion; their function is not to prescribe certainties, but to support the subjective appropriation of knowledge, awaken personal responsibility, and bear witness, with humility, to the other’s process of self-formation^18^,^25^,^29^. This dual communicative and existential demand sketches what Kierkegaard termed reduplication: it is not sufficient to articulate ethical discourses; rather, the mentors’ very act must project into reality the values they seek to embody^27^. In this sense, knowledge is offered not as imposition, but as a capacity; and mentoring is configured as a respectful presence, attentive to the other’s singularity without negating their autonomy^28^.

Nonetheless, in the context of nursing higher education in Mexico, this formative context is shaped by structural factors. As shown by the prior empirical research on which this reflection is based^12^, the absence of clear guidelines on mentoring competencies, institutional ambiguity regarding the mentor’s role, and the lack of systematic training in pedagogical, communicative, and affective skills limit the scope for meaningful mentoring^30^-^32^. Empirical data indicate that, of the 117 educators surveyed, only 27 met the criteria to perform mentoring functions systematically, a figure that reflects the gap between policy and actual practice. Moreover, competencies such as planning, empathic communication, and mentoring guidance were found to correlate negatively with perceived difficulties, suggesting that deficits in these areas intensify the barriers to implementing meaningful mentoring^12^.

When mentoring is conceived as an administrative obligation not recognized within academic incentive systems, or when it lacks support from rigorous training processes, the meaning educators can ascribe to it is weakened^33^,^34^. The risk is clear: mentoring may become an empty practice, disconnected from both the formative ideal and the existential commitment that underpins it. The meaning of mentoring per se does not arise in the abstract, but at the intersection of the educator’s ethical commitment and the institutional and material conditions that make its enactment possible.

Therefore, fostering meaningful mentoring requires a dual strategy: on the one hand, cultivating in faculty a critical awareness that understands mentoring as an ethical and formative act; on the other, intervening structurally in the policies that frame mentoring, so that its enactment is recognized, valued, and supported.

**Teaching competencies as a condition of possibility for meaningful mentoring**

As discussed throughout this reflection, the ethical and existential demands entailed by academic mentoring in nursing, grounded in both Habermas’s communicative ideal and Kierkegaard’s notion of indirect guidance^17^,^19^, can only be realized in and through teaching practice. The deeper meaning of this pedagogical activity is not established in the mere abstraction of its aims, but in the educator’s effective capacity, as a mentor, to mobilize—reflectively and in context—a complex constellation of pedagogical, communicative, and ethical competencies. Mentoring, therefore, cannot be reduced to a technical intervention; rather, it is constituted as a formative practice mediated by conditions of possibility that shape its human and transformative depth.

A first condition is the presence of a pedagogical foundation; without it, the transition from disciplinary expertise to the critical structuring of the educational process becomes impracticable. As noted by Zabalza^30^ and Perrenoud^35^, mentoring requires competencies in deliberate planning, the design of differentiated strategies, formative assessment, and the articulation of theory and practice. In nursing education, this latter condition entails bridging the epistemological gap between clinical knowledge and the lived experience of care^36^; and in the absence of this foundation, mentoring degenerates into didactic improvisation, precluding the emancipatory dialogue proposed by Habermas and preventing the existential openness suggested by Kierkegaard^17^.

Even more decisive is the mentor’s capacity to inhabit the educational setting relationally; communicative competencies entail an ethics of encounter, active listening, empathy, and the recognition of the other as a legitimate interlocutor^37^. Mentoring thus becomes an intersubjective space in which guidance, mediation, and the promotion of active learning^38^,^39^ are intertwined as a practice of epistemic hospitality; the mentor ceases to be a mere transmitter of content and becomes a dialogic facilitator, relinquishing any monopoly over meaning and thereby enabling the student’s subjective development^25^,^18^.

To this dimension is added the value of transversal competencies, understood as dispositions that enable an ethical engagement with complexity; beyond the technical or cognitive, the literature highlights formative attitudes such as social responsibility, creativity, emotional intelligence, metacognition, and a collaborative disposition^39^,^40^. Such competencies are not merely complementary attributes, but constitute the educator’s ethos, their way of being and engaging in education. In this sense, ethical commitment does not appear as an external addition, but as the very condition of possibility for meaningful mentoring. Following Kierkegaard, this entails a“reduplication”: the mentor bears witness, through their actions, to what they seek to elicit, embodying in their own being the call they issued to the other^17^,^27^.

Meaningful mentoring is not a utopia; rather, it is a possibility realized in the integrity of those who, through speech, gesture, and listening, choose to support the development of others as an act of social responsibility.

## Conclusions

Academic mentoring is not merely an instrumental pedagogical resource, but a formative act imbued with ethical, communicative, and existential depth. By placing it in dialogue with the contributions of Habermas and Kierkegaard, its human depth is brought into view, shifting it from a technical-administrative domain to one in which speech, presence, and care emerge as core sources of meaning. The mentor, therefore, is not reduced to a manager of academic trajectories, but is constituted as an agent of epistemic hospitality and ethical responsibility, a mediator between disciplinary knowledge and subjectivity in formation^12^.

In this way, mentoring is revealed as an integrative praxis in which Habermas’s communicative rationality—oriented toward mutual understanding, intersubjective recognition, and the construction of consensus—finds its ethical fulfillment in the free and responsible subjectivity proposed by Kierkegaard. Both frameworks thus converge in an understanding of mentoring as a formative space in which dialogue not only structures learning, but also calls the subject to reflect on their way of being as a professional, articulating competence, freedom, and responsibility in the practice of care. However, this formative conception encounters the paradox of institutions that call for meaningful mentoring while providing limited policy, educational, and symbolic support for its enactment. In this context, the pillars of education proposed by Delors^9^ in articulation with Habermasian communicative rationality and Kierkegaardian existential freedom make it possible to reconceptualize mentoring as a space in which knowledge is offered not as imposition, but as a possibility that requires educators to embody, in their practice, the values they seek to convey.
